# High fat, high sucrose diet promotes increased expression of ACE2 receptor in the SIV-infected host: implications for SARS-CoV-2 infection

**DOI:** 10.3389/fnut.2024.1458106

**Published:** 2024-09-30

**Authors:** Elizabeth C. Delery, Danielle E. Levitt, Angela M. Amedee, Patricia E. Molina, Liz Simon

**Affiliations:** ^1^Comprehensive Alcohol-HIV/AIDS Research Center, Louisiana State University Health Sciences Center, New Orleans, LA, United States; ^2^Department of Physiology, Louisiana State University Health Sciences Center, New Orleans, LA, United States; ^3^Marian University Wood College of Osteopathic Medicine, Indianapolis, IN, United States; ^4^Department of Kinesiology and Sport Management, Texas Tech University, Lubbock, TX, United States; ^5^Department of Microbiology, Immunology, and Parasitology, Louisiana State University Health Sciences Center, New Orleans, LA, United States

**Keywords:** COVID-19, HIV, diet quality, alcohol, risk factor, comorbidities

## Abstract

**Introduction:**

People with pre-existing conditions, including metabolic comorbidities, are at greater risk for complications of SARS-CoV-2 infection and expression of machinery required for viral entry into host cells may be a contributing factor. This study tested the hypothesis that high fat, high sucrose diet (HFSD) and alcohol use increase expression of angiotensin converting enzyme 2 (ACE2) receptor and transmembrane serine protease 2 (TMPRSS2) in tissues isolated from simian immunodeficiency virus (SIV) infected macaques, the most clinically relevant model for the study of HIV.

**Methods:**

Biospecimens obtained from a longitudinal study of SIV-infected, antiretroviral therapy (ART)-treated female rhesus macaques (*Macaca mulatta*) were used to determine whether HFSD and chronic binge alcohol (CBA) increased ACE2 and TMPRSS2 protein and gene expression. Macaques (n = 10) were assigned to HFSD or standard diet (SD) for 3 months before CBA or vehicle administration. Three months later, macaques were infected with SIV; ART was initiated 2.5 months thereafter. Tissue samples including lung, pancreas, and kidney were collected at study endpoint (12 months post-SIV infection).

**Results:**

Protein expression of ACE2 in the lung, whole pancreas, and pancreatic islets was significantly greater in HFSD- than SD-fed macaques with no significant differences in protein expression of TMPRSS2 or mRNA expression of ACE2 or TMPRSS2. CBA did not significantly alter any measures.

**Discussion:**

The increased ACE2 receptor expression observed in lung and pancreas of SIV-infected HFSD-fed female rhesus macaques aligns with reports that diet may increase susceptibility to COVID-19. These data provide direct evidence for a link between dietary quality and cellular adaptations that may increase the risk for SARS-CoV-2 infection.

## 1 Introduction

Severe acute respiratory coronavirus-2 (SARS-CoV-2), the virus that causes coronavirus disease 19 (COVID-19), is the deadliest pandemic of the last 100 years. In the United States alone, over 103 million cases have been reported with over one million deaths as of April 2024 (1). While the primary clinical manifestation is pulmonary disease capable of producing pneumonia and, in severe cases, acute respiratory distress syndrome (ARDS) (2), there is strong evidence demonstrating that SARS-CoV-2 causes multi-organ injury including injury to the heart and blood vessels, lungs, pancreas, kidneys, and brain (3–10). Despite the creation of multiple vaccines and antivirals for use against SARS-CoV-2, in January 2024, the United States was still reporting roughly 1,500 deaths per week from the virus (11).

The US Centers for Disease Control and Prevention reports that risk for severe disease increases in people with pre-existing health conditions including, but not limited to, obesity, type 2 diabetes mellitus (T2DM), and hypertension (12). Nirmatrelvir/ritonavir, an antiviral combination medication to treat COVID-19, is only FDA approved in adults with a high risk of severe disease, including people with a BMI >40 or pre-existing health conditions like heart disease and diabetes (13). Additionally, vaccine effectiveness against SARS-CoV-2 is decreased in people with obesity (14) and in those with T2DM (15). Diet is a modifiable risk factor for metabolic syndromes (16), and a healthy diet can prevent or mitigate metabolic comorbidities, like obesity and T2DM (17, 18).

People with HIV (PWH) might also be at an increased risk for severe illness (12). Literature from the early period of the pandemic did not reveal an association between HIV status and COVID-19 prognosis (19); however, later studies indicated that PWH were more likely to die from COVID-19 complications, even those on ART who were virally suppressed (20–22). Additionally, reduced vaccine efficacy and increased waning of vaccine efficacy is reported in PWH (22). Moreover, alcohol misuse is nearly twice as prevalent among PWH compared to the general population (23) and is a risk factor for HIV infection (24, 25). Alcohol misuse is associated with multisystemic pathophysiological complications (26), including effects on the lung (27), pancreas (28), and kidney (29), organs also affected by SARS-CoV-2. PWH are also at increased risk for obesity-related conditions such as T2DM and kidney disease (30–32), and alcohol misuse increases the risk of dysglycemia among PWH (33). PWH, especially women, commonly have lower dietary quality than the general population (34). Moreover, data from our New Orleans Alcohol Use in HIV (NOAH) study cohort show that PWH with heavy alcohol use have a higher caloric intake, and those that engage in binge and heavy drinking have a higher intake of total and saturated fat (35). Altogether, poor dietary quality and alcohol misuse are prevalent among PWH and are modifiable risk factors associated with comorbidities that increase risk for susceptibility to SARS-CoV-2 infection and severe COVID-19 illness.

The SARS-CoV-2 spike protein binds to the angiotensin converting enzyme 2 (ACE2) receptor of host cells, followed by cleavage of the spike protein by transmembrane serine protease 2 (TMPRSS2) allowing for viral entry into the cell (4, 6). Under normal physiological conditions, ACE2 modulates the renin-angiotensin-aldosterone system (RAAS) which is responsible for homeostatic regulation of vascular function (e.g., blood pressure) (36). In addition to the nasopharyngeal tract and lungs, ACE2 is also expressed in the islets and acinar cells of the pancreas and on the apical surface of the proximal tubules in the kidneys (37). It has been hypothesized that physiological distribution of ACE2 could explain the multisystemic symptoms (3) of COVID-19. There is recent evidence of increased *Ace2* mRNA expression observed in the gastrointestinal tract of mice fed high fat diets (38) and in the lung, kidney, liver, and small intestine, but not the heart, of chronic alcohol-administered rats (39). Together, these published data show the potential for diet and alcohol to modify expression of the viral entry protein ACE2 at the mRNA level.

Although its role in SARS-CoV-2 cell entry is known, less is known about the role of TMPRSS2 in normal physiology (40). High fat-fed mice had decreased *Tmprss2* mRNA expression in the gastrointestinal tract (38). In contrast, obese mice had increased *Tmprss2* expression in the trachea but not in the lung compared to lean mice (41). Moreover, Tmprss2 protein expression was increased in the lung of rats fed a diet high in fat, with or without high sucrose (42). However, chronic alcohol administration did not affect *Tmprss2* mRNA expression in rat lung, kidney, liver, small intestine, or heart; However, protein expression was not examined (39). Together, these data indicate that diet-mediated alterations in *TMPRSS2* could be organ-specific, and protein-level data are sparse.

It is possible that the diet and alcohol use patterns that contribute to comorbid metabolic conditions are also modifiable risk factors that increase expression of proteins required for SARS-CoV-2 cell entry (e.g., ACE2 and TMPRSS2). Therefore, using a rhesus macaque model, we tested the hypothesis that a high fat, high sucrose diet (HFSD) and chronic binge alcohol (CBA) administration contribute to increased expression of ACE2 and TMPRSS2 in the lungs, pancreas, and kidneys in the context of simian immunodeficiency virus (SIV) infection.

## 2 Materials and methods

### 2.1 Non-human primate study design

Retrospective tissue samples were obtained from a subset of animals included in a parent longitudinal study that was designed to determine the impact of alcohol and diet on susceptibility to SIV infection and disease progression. All animal experiments were approved by the Institutional Animal Care and Use Committee at Louisiana State University Health Sciences Center (LSUHSC) in New Orleans, Louisiana, and adhered to the “NIH Guide for the Care and Use of Laboratory Animals” (National Research Council, National Academic Press, Washington, DC, USA, 1996). Adult (6–9 years old) female rhesus macaques (*Macaca mulatta*; n = 10) were assigned to HFSD (Primate Diet TD.07802, protein/fat/carbohydrates 16/42/42% of total kcal and 27% sucrose by weight, Envigo Teklad Diets, Madison, WI) or standard diet (SD; Teklad Global 20% Protein Primate Diet 2050, protein/fat/carbohydrates 29/14/57% of total kcal, Envigo Teklad Diets). Three months later, daily binge alcohol (CBA, *n* = 6; 50–60 mM peak blood alcohol, 5 days/week) or isovolumetric water (VEH) administration via 30-min intragastric infusions was initiated. The HFSD group (*n* = 5) was a small pilot sample where 3 and 2 macaques were assigned to receive CBA and VEH, respectively, and samples from 5 SD-fed animals were matched on alcohol group assignment (CBA or VEH). The macaques were randomized into their respective groups based on body weights, *in vitro* kinetics of viral loads in peripheral blood mononuclear cells, and genotypes (MHC typing). Three months after initiating CBA or VEH, macaques were infected with SIV_mac251_ virus (both groups) and SIV_17E − Fr_ (HFSD group) and daily antiretroviral therapy [ART; SD group: emtricitabine, 30 mg/kg and tenofovir, 20 mg/kg; HFSD group: Biktarvy (bictegravir 6 mg/kg, emtricitabine 30 mg/kg and tenofovir alafenamide 4 mg/kg); drugs were a generous gift from Gilead Sciences Inc. (Foster City, CA)] began 2.5 months thereafter. We have previously published that this regimen of ART successfully suppresses viral load without overt adverse side effects (43). Blood was collected weekly throughout SIV infection and at necropsy for the routine measurement of plasma viral load (44), and the final 6 plasma viral load measures, including day of necropsy, were averaged for each macaque.

### 2.2 Necropsy and tissue collection

Macaques were humanely euthanized 9 months after ART initiation using the standards set forth by the Office of Laboratory Animal Welfare (OLAW) and tissue samples including lung, pancreas, and kidney collected. A portion of each tissue was flash frozen in liquid nitrogen and stored at −80°C until RNA extraction. Another portion of each tissue was fixed in zinc-buffered formalin for paraffin embedding.

### 2.3 Immunohistochemistry

Sections of formalin-fixed, paraffin-embedded lung, pancreas, and kidney were sliced at 5 μm, mounted two per slide, and subsequently stained using previously published methodology (45). For a given tissue (i.e., lung, pancreas, and kidney), all slides were processed simultaneously. In brief, the slides were dried in a 60°C oven, then deparaffinized with washes of xylene, rehydrated in 100%, 95%, and 80% ethanol, before antigen unmasking in low pH citrate buffer (0.1M; pH 6) diluted 1:100 in deionized water. Slides were permeabilized with phosphate buffered saline (PBS) containing 0.2% fish skin gelatin (FSG; Millipore Sigma, Burlington, MA) and 0.1% Triton-X 100 (Millipore Sigma), then blocked with 10% normal donkey serum (NDS; D9663, Sigma, St. Louis, MO) for 1 h at room temperature in a humidified black box and incubated with TMPRSS2 primary antibody (Anti-TMPRSS2 mouse IgG, 1:200; #sc-515727, Santa Cruz Biotechnology, Dallas, TX) diluted in 10% NDS for 1 h at room temperature. The slides were washed with PBS-FSG and incubated in secondary antibody (AlexaFluor 488 donkey anti-mouse IgG, 1:500; #ab150109, Abcam, Cambridge, MA) diluted in PBS-FSG for 1 hour at room temperature. The slides were washed with PBS-FSG before incubating with ACE2 primary antibody (Anti-ACE2 rabbit IgG, 1:500; #ab15348, Abcam) diluted in 10% NDS for 1 h, washed with PBS-FSG, and then incubated in secondary (AlexaFluor 555 donkey anti-rabbit IgG, 1:500; #A31572, Invitrogen, Carlsbad, CA) diluted in PBS-FSG for 1 h before slides were washed and mounted with Vectashield Hardset mounting media with DAPI (Vector Labs, Burlingame, CA). Primary antibody-only and secondary antibody-only control experiments were performed prior to formal experimentation. The researchers who performed immunohistochemistry were blind to group assignments until quantification was complete.

### 2.4 Quantification of TMPRSS2 and ACE2 using corrected total cell fluorescence

All slides were imaged at the same exposure settings using the Nikon TE2000U fluorescent microscope. For analysis, 15 random images were taken at 20x magnification from each section of tissue per slide (2 per slide, per animal). FIJI ImageJ (46) was used to collect image data on the integrated density, area of each image, and the mean fluorescence intensity (MFI) of the background. Images were normalized to each other by setting threshhold values in ImageJ (NIH, Bethesda, MD, USA). The corrected total cell fluorescence (CTCF), accounting for background MFI, was calculated using the equation (47):

(1) $$\begin{array}{l} \text{CTCF}=\text{Integrated Density}-(\text{Area of selected cell}\\ \times \text{MFI of background}) \end{array}$$

To assess islet and proximal convoluted tubule expression of ACE2, the freehand trace tool was used to circle either the islets or the proximal convoluted tubules and the integrated density, area of the traced shape, and mean intensity of the background was collected to calculate the CTCF using the previously mentioned equation. This standardized methodology has previously been used to correct for potential variability in images obtained from immunohistochemistry experiments to accurately quantify protein expression in complex tissues (48). Moreover, the use of quantitative immunofluorescence to assess protein expression has been validated against mass spectrometry (49).

### 2.5 RNA isolation and quantitative real-time polymerase chain reaction (qPCR)

To assess *ACE2* and *TMPRSS2* mRNA expression, 600 ul of lysis buffer RLT (Qiagen, Valencia, CA) containing 1% β-mercaptoethanol was added to frozen tissue samples (~30 mg; lung, pancreas, and kidney) and total RNA was extracted using the RNeasy Mini Kit (Qiagen) according to manufacturer's instructions. cDNA was synthesized from 2 μg of RNA using the Quantitect reverse transcription kit (Qiagen) in 40 uL final reaction volume according to manufacturer's instructions. Custom primers designed to span exon-exon junctions were purchased from Integrated DNA Technologies (Coralville, IA; Table 1). Final reactions contained cDNA (50 ng), primers (500 nM), SyBr green (Quantitect SyBr Green PCR kit, Qiagen), and nuclease-free water to 20 uL. qPCR reactions were carried out in duplicate using a CFX96 thermal cycler (Bio-Rad, Hercules, CA) with ribosomal protein S13 (*RPS13*) as the endogenous control for mRNA assessment as previously validated (50) and reported (51–54) by our laboratory. The researchers who performed qPCR were blind to group assignments until quantification was complete.

**Table 1:** List of primers for qPCR analysis (primers from IDT technologies).

**Gene**	**Forward primer**	**Reverse primer**
Angiotensin converting enzyme 2 receptor *(ACE2)*	TGAATGCCTACCCTTCCT	GGGAACTGTCAAAGAGTACAG
Transmembrane serine protease 2 *(TMPRSS2)*	GGATGGTGGCTGGAAATAAA	AGGGCACTGTCTACATTCT
Ribosomal protein S13 *(RPS13)*	CCCACTTGGTTGAAGTTGA	CAGGATCACACCGATTTGT

### 2.6 Statistical analyses

Statistical analyses were performed using GraphPad Prism 9.0.0 (GraphPad Software, San Diego, CA). Viral load data were log-transformed prior to analyses. Data were checked for outliers using Grubbs' test and for normality using the Kolmogorov-Smirnov test and QQ plots. Grubbs' test identified an extreme outlier for ACE2 and TMPRSS2 expression in the lung; the corresponding values were removed prior to analyses. Raw data for ACE2 mRNA expression in the kidney were log-transformed prior to analysis to correct for the violation of normality. Data were analyzed using a diet group assignment (SD, *n* = 5; HFSD, *n* = 5) × alcohol group assignment (VEH, *n* = 4; CBA, *n* = 6) ANOVA. Because of the small sample size in this pilot study and the primary goal to assess differences in protein and mRNA expression of ACE2 and TMPRSS2 between levels of each factor, we used main effects only models. Cohen's d effect sizes between groups are reported where statistically significant main effects exist or as otherwise indicated. Data are presented as mean ± SEM. P-values provided in the text are exact, whereas those shown in figures use standard conventions (e.g., ^*^p < 0.05, ^**^p < 0.01, etc.).

## 3 Results

### 3.1 ACE2 and TMPRSS2 protein expression

**Figure 1:**
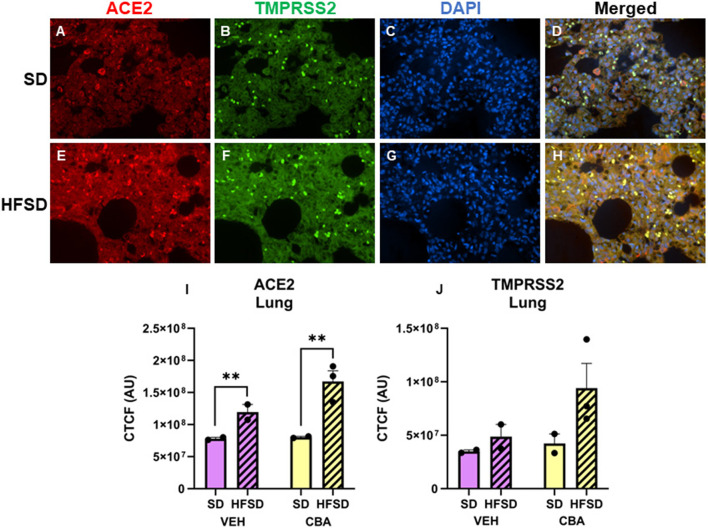
Representative images of lung stained for ACE2 (red), TMPRSS2 (green), DAPI (blue), and merged for the standard diet [SD; **(A–D)**] and high fat, high sucrose diet [HFSD; **(E–H)**] groups. Images are shown at 40x magnification; merged images show protein expression patterns. Expression (corrected total cell fluorescence, CTCF) of ACE2 **(I)** and TMPRSS2 **(J)** protein in the lung for the SD and HFSD groups in the vehicle (VEH)- or chronic binge alcohol (CBA)-administration conditions. ^**^p < 0.01 (main effect of diet). Analyzed by 2-way ANOVA; M±SEM.

Representative images of immunostained lung are shown in Figures 1A–H. Protein expression of ACE2 in the lung was greater in the HFSD group than the SD group (*p* = 0.005; d = 2.65, large effect size; Figure 1I). No significant differences in protein expression of TMPRSS2 were observed in the lung between HFSD- and SD-fed groups (Figure 1J), nor were there any significant differences in protein expression of ACE2 or TMPRSS2 between VEH and CBA groups.

**Figure 2:**
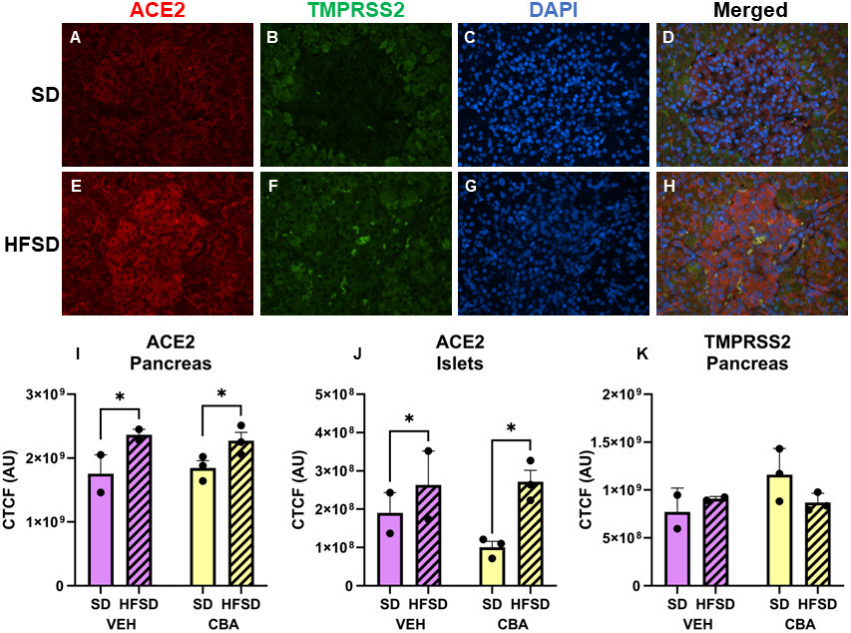
Representative images of pancreas stained for ACE2, TMPRSS2, DAPI, and merged for the standard diet [SD; **(A–D)**] and high fat, high sucrose diet [HFSD; **(E–H)**] groups. Images are shown at 40x magnification; merged images show protein expression patterns. Expression (corrected total cell fluorescence, CTCF) of ACE2 in the whole pancreas **(I)** and islets **(J)** and of TMPRSS2 protein in the whole pancreas **(K)** for the SD and HFSD groups in the vehicle (VEH)- or chronic binge alcohol (CBA)-administration conditions. *p < 0.05 (main effect of diet). Analyzed by 2-way ANOVA; M±SEM.

Representative images of immunostained pancreas are shown in Figures 2A–H. Protein expression of ACE2 in the pancreas (endocrine and exocrine portions) was greater in the HFSD group than the SD group (*p* = 0.012; d = 2.30, large effect size; Figure 2I). We further assessed ACE2 expression in the pancreatic islets. Expression was higher in the HFSD group than the SD group (*p* = 0.020; d = 1.92, large effect size; Figure 2J). No significant differences in protein expression of TMPRSS2 were observed in the pancreas between HFSD- and SD-fed groups (Figure 2K), nor were there any significant differences in protein expression of ACE2 or TMPRSS2 between VEH and CBA groups.

**Figure 3:**
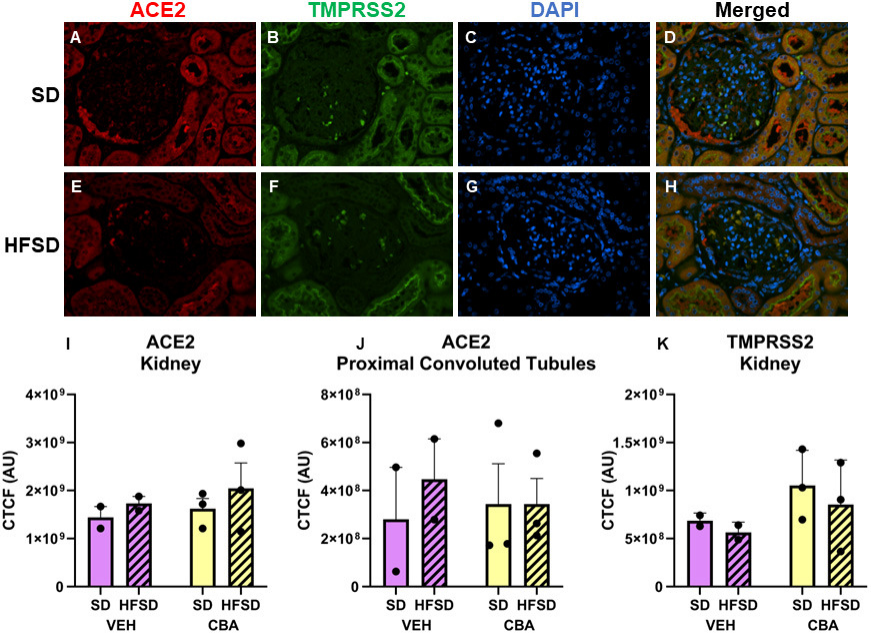
Representative images of kidney stained for ACE2, TMPRSS2, DAPI, and merged for the standard diet [SD; **(A–D)**] and high fat, high sucrose diet [HFSD; **(E–H)**] groups. Images are shown at 40x magnification; merged images show protein expression patterns. Expression (corrected total cell fluorescence, CTCF) of ACE2 in the whole kidney **(I)** and proximal convoluted tubules **(J)** and of TMPRSS2 protein in the whole kidney **(K)** for the SD and HFSD groups in the vehicle (VEH)- or chronic binge alcohol (CBA)-administration conditions. Analyzed by 2-way ANOVA; M±SEM.

Representative images of immunostained kidney are shown in Figures 3A–H. No significant differences in ACE2 expression in the kidney (convoluted tubules plus glomeruli; Figure 3I) or proximal convoluted tubules alone (Figure 3J) were observed between HFSD- and SD-fed groups or between VEH and CBA groups. No significant differences in TMPRSS2 protein expression were observed in the kidney between HFSD- and SD-fed groups (Figure 3K), nor were there any significant differences in protein expression of ACE2 or TMPRSS2 between VEH and CBA groups.

### 3.2 ACE2 and TMPRSS2 mRNA expression

**Figure 4:**
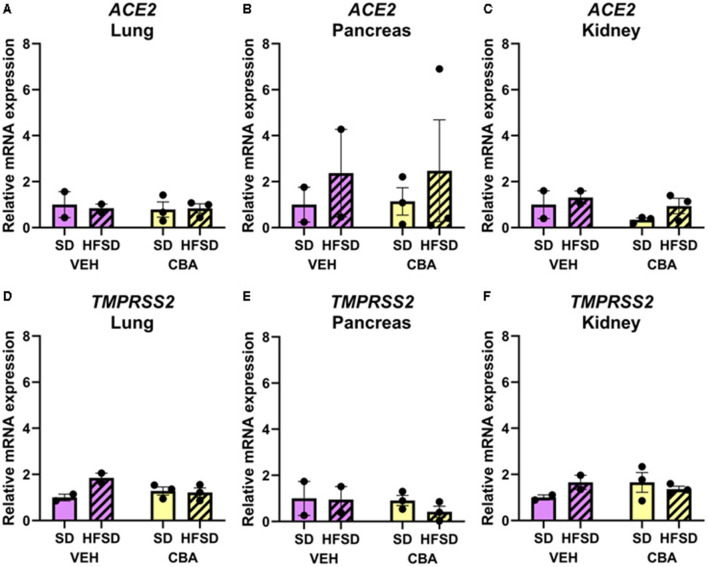
Expression of ACE2 mRNA in the lung **(A)**, pancreas **(B)**, and kidney **(C)**, and TMPRSS2 mRNA in the lung **(D)**, pancreas **(E)**, and kidney **(F)** relative to *RPS13* for the standard diet (SD) and high fat, high sucrose diet (HSFD) groups in the vehicle (VEH)- or chronic binge alcohol (CBA)-administration conditions. Data for ACE2 mRNA expression in the kidney were log10 transformed prior to analysis to correct for the violation of normality; raw data are presented. Analyzed by 2-way ANOVA; M±SEM.

No significant differences in *ACE2* (Figures 4A–C) or *TMPRSS2* (Figures 4D–F) mRNA expression were observed between HFSD and SD groups or between VEH and CBA groups in the lung, pancreas, or kidney.

### 3.3 Viral load

**Figure 5:**
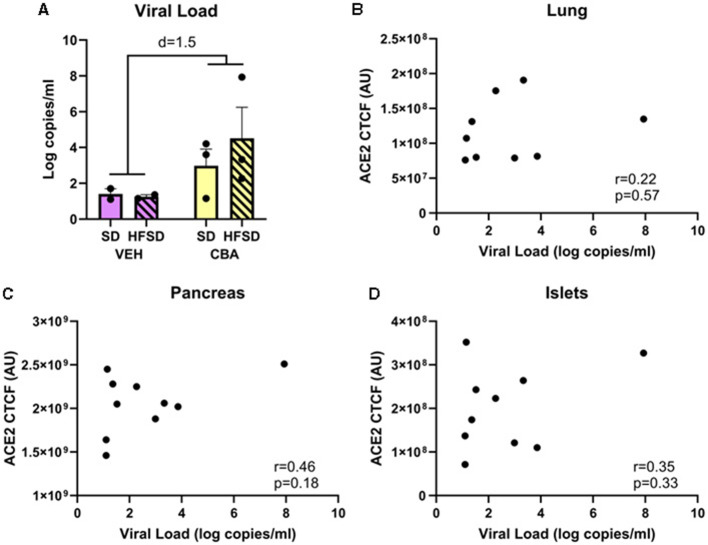
Viral load **(A)** for the SD and HFSD groups in the vehicle (VEH)- or chronic binge alcohol (CBA)-administration conditions, analyzed by 2-way ANOVA; M±SEM. *A posteriori* Pearson correlations between ACE2 receptor protein expression and viral load in the lung **(B)**, pancreas **(C)**, and islets **(D)**.

The average of the final six plasma SIV levels, including on the day of necropsy, were not significantly different between the SD and HFSD groups or between the CBA and VEH groups in this subset of animals. However, there was a trend with a large effect size for CBA to increase viral load in this subset of animals (mean ± SEM; VEH: 1.3 ± 0.1 log copies per ml; CBA 3.7 ± 0.9 log copies per ml; Cohen's d = 1.5; Figure 5A). *A posteriori* correlations between the levels of ACE2 expression in lung, pancreas, and islets and levels of viremia were calculated, and no significant correlation was observed (lung: r = 0.22, R^2^ = 0.05, *p* = 0.57; pancreas: r = 0.46, R^2^ = 0.21, *p* = 0.18; islets: r = 0.35, R^2^ = 0.12, *p* = 0.33; Figures 5B–D).

## 4 Discussion

In the present study, increased expression of the ACE2 receptor was observed in the lung and pancreas, but not the kidneys, of SIV-infected female rhesus macaques fed a high fat, high sucrose diet. Because ACE2 receptor is required for SARS-CoV-2 cell entry, our study provides evidence that diet may increase susceptibility to COVID-19 and associated pulmonary and pancreatic complications.

Pulmonary complications are common in patients with COVID-19 (2, 7, 10) and these may progress to prolonged dyspnea secondary to pulmonary vascular injury and fibrosis in the post-COVID syndrome known as long COVID (55). A greater proportion of obese patients with COVID-19 in the hospital setting experience ARDS and require mechanical ventilation (56–58) than patients with normal body mass index (BMI). Poor dietary quality, including refined carbohydrates and energy-dense food intake, is a determinant of obesity (59–61). We observed that HFSD fed rhesus macaques had increased ACE2 expression in the lungs. Similarly, a previous study reported that ACE2 protein but not mRNA expression was increased in the lungs of patients with T2DM, an obesity-related condition (62). Therefore, poor dietary quality may increase risk for SARS-CoV-2 cell entry and thus may partially underlie the increased risk for severe pulmonary complications of COVID-19 in patients with obesity with poor dietary quality, especially those with HIV. However, further mechanistic examination is warranted.

Beta cells express ACE2, and elevated *Ace2* expression has been observed in a genetic rat model of T2DM (39). Moreover, in otherwise healthy older rhesus macaques, the SARS-CoV-2 virus infected beta cells and induced metabolic abnormalities characterized by beta cell transdifferentiation and fibrosis, decreased insulin production, and impaired glycemic control; beta cell impairment was less severe in younger adult macaques (63). These data strongly support the connection between the results of the present study showing increased ACE2 receptor expression with a diet high in fat and sucrose, and potential facilitation of SARS-CoV-2 viral entry leading to endocrine pancreatic sequelae of COVID-19.

SARS-CoV-2 infection can also have consequences for the exocrine pancreas. In a study of over 300 patients with COVID-19 pneumonia, 1 in 10 patients reported acute pancreatitis, and the rate increased to 1 in 3 among critically ill patients (64). Direct exocrine pancreatic injury arises from SARS-CoV-2 cell entry via ACE2 receptors on the acinar cells (65). It is reported that ACE2 protein levels vary more than mRNA, indicating some form of post-transcriptional regulation (3, 37, 65). Similarly, we observed a HFSD-mediated increase in protein but not mRNA expression of ACE2 in the lungs and pancreas, but the specific mechanism of post-transcriptional regulation is unknown.

While there was no statistically significant difference in plasma SIV levels between groups in this subset of animals, there was a trend for higher viral load among CBA-administered macaques, which is consistent with previous observations (66). Since the relationship between SIV and ACE2 expression has not been assessed, the lack of difference in SIV levels between the HFSD and SD groups provide support that viral loads did not drive the differences in ACE2 expression between diet groups. Furthermore, there were no differences in ACE2 between alcohol conditions, whereas SIV levels tended to be higher in the CBA group. We also examined the relationship between viral load and ACE2 receptor expression in lung, pancreas, and pancreatic islets; no correlation was observed. Therefore, it is unlikely that differences in viral load accounted for the increased ACE2 receptor expression with HFSD in the present study. However, the increased ACE2 expression in lung and pancreas observed in the HFSD group in the present study indicates that diet quality may affect susceptibility of these tissues to SARS-CoV-2 infection in people with HIV.

The results of this study provide a critical link between diet and ACE2 expression; however, the study is not without limitations. The HFSD group was on an ART regimen that included bictegravir in combination with tenofovir and emtricitabine (Biktarvy), whereas the SD group received tenofovir and emtricitabine alone. Therefore, we cannot discount the possibility for the difference in ART regimen to have partially contributed to increased lung and pancreas ACE2 receptor expression in the HFSD group. Although bictegravir has been examined using computational approaches as a potential drug to repurpose for the treatment of SARS-CoV-2 infection (67), no published work has linked bictegravir with alterations of ACE2 receptor expression. Therefore, it is highly unlikely that Biktarvy accounted for the diet-related differences in ACE2 receptor expression in the present study. Moreover, all macaques in this study were female, so the results cannot be extended to males. While our results demonstrate that ACE2 receptor expression was greater in HFSD-fed, SIV-infected, ART-treated female macaques, the underlying mechanisms remain to be elucidated, and an examination of direct effects (e.g., *in vitro*) of HFSD on ACE2 receptor expression in isolated lung and pancreas cell types is warranted. We were also unable to perform infectivity experiments with SARS-CoV-2 to verify whether increased ACE2 receptor expression observed on cells in the lung and pancreas resulted in facilitation of viral entry; this will be examined in future work. We did not find differences in TMPRSS2 expression at the gene or protein level; however, other proteases involved in SARS-CoV-2 pathogenesis, including cathepsin L (68), were not assessed. Finally, our small sample size precluded the investigation of a possible alcohol-mediated exacerbation of diet-mediated changes in ACE2 expression and should be examined in future studies.

## 5 Conclusions

These data provide direct evidence for a link between dietary quality and cellular adaptations that may increase the risk for SARS-CoV-2 infection in the context of SIV/HIV infection, urging diet counseling and increased access to higher-quality foods in this population. With the continuing evolution of the SARS-CoV-2 virus and new variants that appear to have increased transmissibility due to increased binding of ACE2 (69–71), understanding if specific comorbid conditions or risk factors increase ACE2 expression would be important to isolate and protect high risk and vulnerable populations from potential exposure.

## Data Availability

The data presented in the study are deposited in the Open Science Framework repository at https://osf.io/8h6ep/?view_only=ee0a426915d64c80b3b89a1218be38af.
